# Dissecting the *miR-451a*-Mif Pathway in Endometriosis Pathophysiology Using a Syngeneic Mouse Model: Temporal Expression of Lesion Mif Receptors, Cd74 and Cxcr4

**DOI:** 10.3390/biomedicines10071699

**Published:** 2022-07-14

**Authors:** Warren B. Nothnick, Amanda Graham

**Affiliations:** 1 Department of Molecular and Integrative Physiology, University of Kansas Medical Center, Kansas City, KS 66160, USA; agraham@kumc.edu; 2 Department of Obstetrics and Gynecology, University of Kansas Medical Center, Kansas City, KS 66160, USA; 3 Center for Reproductive Sciences, Institute for Reproduction and Perinatal Research, University of Kansas Medical Center, Kansas City, KS 66160, USA

**Keywords:** endometriosis, experimental model, *miR-451a*, MIF, CD74

## Abstract

Endometriosis is an enigmatic disease characterized by pain and infertility in which endometrial tissue grows in ectopic locations, predominantly the pelvic cavity. The pathogenesis and pathophysiology of endometriosis is complex and postulated to involve alterations in inflammatory, cell proliferation and post-transcriptional regulatory pathways among others. Our understanding on the pathogenesis and pathophysiology of endometriosis is further complicated by the fact that endometriosis can only be diagnosed by laparoscopy only after the disease has manifested. This makes it difficult to understand the true pathogenesis as a cause-and-effect relationship is difficult to ascertain. To aid in our understanding on endometriosis pathogenesis and pathophysiology, numerous rodent models have been developed. In this case, we discuss further assessment of a *miR-451a*—macrophage migration inhibitory factor (Mif) pathway which contributes to lesion survival. Specifically, we evaluate the temporal expression of lesion Mif receptors, *Cd74* and *Cxcr4* using host mice which express wild-type or *miR-451a* deficient lesions. Similar to that observed in humans and a non-human primate model of endometriosis, *Cd74* expression is elevated in lesion tissue in a temporal fashion while that of *Cxcr4* shows minimal increase during initial lesion establishment but is reduced later during the lifespan. Absence of *miR-451a* during initial lesion establishment is associated with an augmentation of *Cd74*, but no *Cxcr4* expression. The data obtained in this study provide further support for a role of Mif receptors, Cd74 and Cxcr4 in the pathophysiology of endometriosis.

## 1. Introduction

### 1.1. Endometriosis Pathogenesis and Pathophysiology

Endometriosis is an estrogen-dependent, chronic, benign gynecological disease in which endometrial glands and stroma establish in the peritoneal cavity. Endometriosis occurs in approximately 10% of women of reproductive age and is characterized by pelvic pain and infertility [1,2]. Several theories and contributing factors have been proposed in the pathogenesis of this disease. For example, accumulating evidence suggests a role for endometrial stem cells, a dysfunctional immune response, genetic predisposition, aberrant peritoneal environment and alterations in the eutopic endometrium [3,4]. Pathological mechanisms are complex and include abnormal hormonal, epigenetic, genetic, and immunologic/inflammatory pathways among others [1,2,3,4]. One of the greatest challenges in understanding the pathogenesis and pathophysiology of endometriosis stems from the fact that the disease can only be diagnosed after it has manifested. This creates a conundrum of cause and effect making it difficult to establish if the altered physiology of lesion tissue is a result of or a contributing factor in lesion establishment and survival. To overcome this issue, experimental models have been developed in rodents as well as non-human primates, with the former providing the advantage of being able to genetically modify the tissue donor or recipient host to dissect the role of specific genes in endometriosis pathophysiology.

### 1.2. Syngeneic Mouse Models to Study Endometriosis Pathophysiology

**Figure 1:**
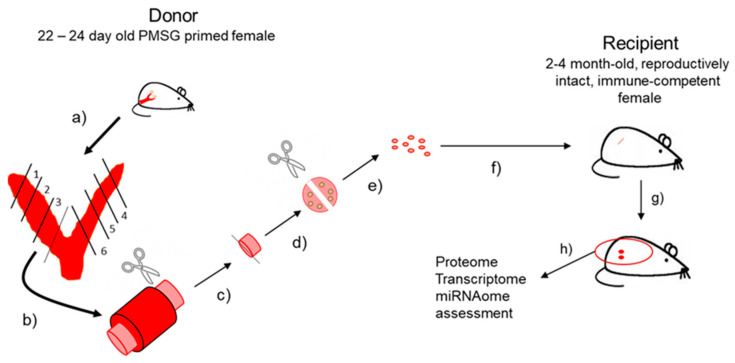
Diagram outlining the procedure for experimental endometriosis induction.

Numerous mouse models have been developed to study endometriosis. Burns and colleagues [5] provide a detailed review of “best fit” experimental mouse models for endometriosis and the reader is referred to this reference for greater detail. As emphasized in that review, for a “best fit” model, the host in which the disease is established should be immunocompetent and hormonally intact. Administration of exogenous estrogen/estradiol (E2) to the host, often used to promote lesion establishment and survival should ideally be avoided to prevent masking or confounding of study endpoints. With this in mind, we developed one of the first immune competent, reproductively intact mouse models for endometriosis study that avoided administration of exogenous E2 to host mice [6]. Figure 1 depicts the general procedure for induction of experimental disease based upon this concept. 

### 1.3. miR-451a-MIF Pathway in Endometriosis Pathophysiology

It is well established that microRNAs (miRNAs) play a role in the pathophysiology of endometriosis reviewed in [7,8,9]. miRNAs are small, non-coding RNAs which function as epigenetic regulators that modulate gene expression post-transcriptionally [10,11]. Cell proliferation, invasion and apoptosis are all events conducive to endometriosis development and survival [12] and miRNAs have been implicated to play a vital role in these events. As a result, researchers have compiled miRNA expression profiles for endometriosis in both the disease tissue and eutopic endometrium as well as control patients [13,14,15,16]. These data were integral in developing our understanding on their potential role in endometriosis pathophysiology which to date continues to emerge. Of the numerous miRNAs mis-expressed in endometriotic tissue, *miR-451a* (originally identified as *miR-451*) is one of the more well-studied miRNAs [17,18,19,20,21,22].

Similar to most miRNAs, *miR-451a* is a putative regulator of hundreds of mRNA transcripts. Perhaps most relevant to the pathophysiology of endometriosis is the putative cytokine target, macrophage migration inhibitory factor (MIF). Endometritoic lesions cells are a rich source of MIF which exhibits mitogenic activity promoting the growth of endothelial cells [23] as well as the ability to stimulate prostaglandin E2, cyclooxygenase-2 [24], vascular endothelial growth factor, interleukin-8 and monocyte chemotactic protein-1 [25]. These cytokines are associated with a proliferative and angiogenic phenotype conducive to endometriotic establishment, growth and survival. In addition, MIF is predominantly expressed in glandular epithelial cells of both eutopic endometrium [26,27] and robustly expressed in epithelium of active and early/stage I endometriotic lesions [27] with focal stromal staining in both tissue types. Two independent studies [6,28] utilized experimental mouse models for endometriosis to demonstrate that Mif plays an active role in endometriosis pathophysiology as administration of the MIF antagonist, ISO-1, reduced endometriotic lesion size.

For MIF to elicit a biological response it must bind with cell surface receptors. CD74 (also referred to as invariant chain) was identified as the first receptor for MIF [29]. CD74 (along with CD44) was demonstrated to be responsible for MIF liberation of angiogenic factors in vitro, ref. [25] and our in vivo studies in humans as well as a baboon model for endometriosis [30] demonstrate that CD74 is robustly expressed in endometriotic lesion tissue. Additionally, C-X-C chemokine receptor type 4 (CXCR4) has been shown to function as a non-canonical MIF receptor in several cell types [31,32,33,34]. With respect to endometriosis, CXCR4 transcript and protein is expressed in human endometriotic lesion tissue [35,36,37] including deep-infiltrating lesions [38], but its association with CD74 has not been assessed in experimental models for endometriosis. Based upon these observations, the objective of the current study was to concurrently assess expression of *Cd74* and *Cxcr4* in endometriotic lesion tissue from mice with experimentally-induced disease to see if we could recapitulate a similar pattern of expression observed in human disease. 

## 2. Materials and Methods

### 2.1. Experimental Mouse Model for Endometriosis

All animal experiments were conducted at the University of Kansas Medical Center under the guidance of Dr. Nothnick following the relevant guidelines and regulations. Experimental procedures incorporating animals were approved by the University of Kansas Medical Center Institutional Animal Care and Use Committee (IACUC). Experimental endometriosis was induced as depicted in Figure 1.

Briefly, donor female mice consisted of 22- to 24-day old C57BL/6 female mice. These females were either wild-type C57BL/6 or females deficient for *miR-451a*/*miR-144-3p*. To stimulate endogenous estrogen production and subsequent estrogenic responses within the uterus, all donor females were injected s.c. with pregnant mare serum gonadotropin (PMSG; 2 IU; Sigma Chemical Company, St. Louis, MO, USA). Uteri were then harvested from donor mice approximately 40–42 h after PMSG administration (Figure 1, step a). Each uterus was cut into 6 equal size fragments (Figure 1, step b) and protruding endometrial tissue (stroma and epithelium) was separated from myometrium with the aid of a dissecting microscope (Figure 1, step c). Protruding endometrial tissue (which contained stromal as well as glandular and luminal epithelium) was first cut into halves (Figure 1, step d) to yield 12 fragments of equal size (1 mm^3^; Figure 1, step e). Uterine fragments were prepared for transfer to recipients (Figure 1, step f) by resuspending in 0.4 mL of sterile saline. Recipient mice (2- to 4-month old wild-type C57BL/6 immuno-competent, reproductively intact females, which express *miR-451a*/*miR-144-3p*) were anesthetized with ketamine/xylazine and an antibiotic ointment was placed over the corneas to avoid corneal abrasions. The area over the right rib cage was prepared for surgery and a small incision (approximately 0.5 cm) was made exposing the peritoneal cavity. Tissue fragments resuspended in PBS were injected into the peritoneal cavity through the incision (Figure 1, step f) and the incision was then closed with wound clips. Carprofen analgesic was given post-operatively at the conclusion of the surgery and again 24 h later. Mice were then sacrificed at indicated time post endometriosis induction (Figure 1, step g) and lesions processed for mRNA isolation in this experiment (Figure 1, step h). Week 0 controls consisted of endometrial fragments which were not transferred to recipient mice and were immediately placed into Trizol (Life Technologies, Carlsbad, CA, USA) and subjected to RNA isolation as described below.

### 2.2. RNA Isolation and qRT-PCR of miRNAs and mRNAs

Endometriotic lesions were harvested at the indicated time points and RNA was isolated from tissue using Trizol (1.0 mL of Trizol/100 mg of tissue) following the protocols provided by the manufacturer. Total RNA was reversed transcribed using 1 µg of total RNA using reverse transcription (RT) kits (Applied Biosystems; Foster City, CA, USA) following the manufacturer’s protocol. Primers for mouse *Cd74* and *Cxcr4* were designed using Primer-Blast and synthesized by Integrated DNA Technology (IDT, Coralville, IA, USA) and sequences are listed in Table 1. *Cd74* and *Cxcr4* expression levels were normalized to 18S rRNA using primers from Applied Biosystems (Table 1).

All qRT-PCR reactions were completed on a QuantStudio7 Flex Real-Time PCR System (Applied Biosystems). All samples were run in triplicate and the average value used in subsequent calculations. The 2-delta-delta CT method was used to calculate the fold-change values among samples as previously described by our group [17,18,20,30]. qRT-PCR intra- and inter-assay coefficients of variation were both less than 6%.

### 2.3. Statistical Analysis

Data were first assessed for normal (Gaussian) distribution. All data failed to exhibit Gaussian distribution and were therefore transformed to log2 scale with analysis conducted as described in each Figure legend. All analysis was conducted using GraphPad Instat3 (GraphPad Software, La Jolla, CA, USA). Significance was set at *p*  <  0.05 for all analyses.

## 3. Results

**Figure 2:**
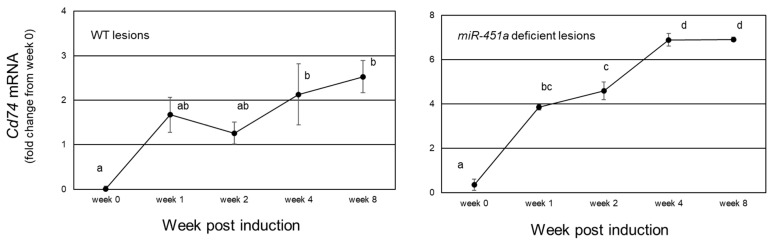
*Cd74* mRNA expression during lesion lifespan. *Cd74* mRNA expression was analyzed in lesion tissue from mice with experimentally induced endometriosis which harbored wild type (WT; right panel) or *miR-451a* deficient lesions (left panel). Data were expressed as log2 values and compared to eutopic tissue which was harvested but not transferred intraperitoneally (week 0). Different letters (a, b, etc.) indicate statistical significance among means by one-way ANOVA and Tukey’s post-hoc comparison. Data are displayed as the mean ± SEM with *n* = 3 to 4 mice/time point.

We first examined endometriotic lesion expression of the cognate Mif receptor, *Cd74* in endometriotic lesion tissue derived from wild-type and *miR-451a*/*miR-144-3p* deficient tissue. *Cd74* mRNA expression increased from 1 through 8 weeks post-endometriosis induction (week 0) but there was high variation among the fold increase in expression. In order, to make variation similar across orders of magnitude, data were log2 transformed and are presented in Figure 2. In mice with wild-type (WT) lesions, *Cd74* expression increased at 1 to 2 weeks post-induction reaching statistically higher levels of expression at weeks 4 and 8 with a maximal increase of 6-fold compared to week 0. When *miR-451a* deficient tissue was used to develop ectopic lesions, there was a more robust up-regulation of *Cd74* expression compared to that of WT lesions. Specifically, *Cd74* expression increased approximately 8-fold, 1 to 2 weeks post-induction, reaching peak levels between weeks 4 and 8 as high as 194-fold above week 0 levels. In these same samples (for the 0-to-4-week timepoints) reduced levels of Mif expression were associated with elevated *miR-451a* expression. Compared to human disease, our mouse model closely replicates the pattern of expression of endometriotic lesion *miR-451a*, *Mif* and *Cd74* expression observed in human endometriotic lesion tissue.

**Figure 3:**
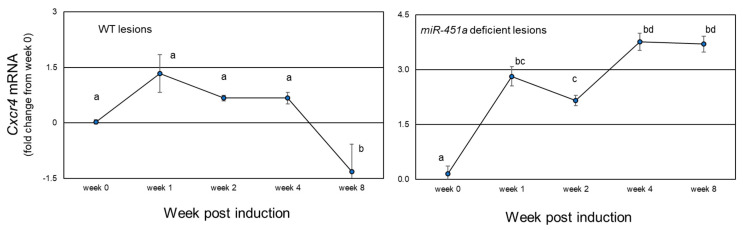
*Cxcr4* mRNA expression during lesion lifespan. *Cxcr4* mRNA expression was analyzed in lesion tissue from mice with experimentally induced endometriosis which harbored wild type (WT; right panel) or *miR-451a* deficient lesions (left panel). Data were expressed as log2 values and compared to eutopic tissue which was harvested but not transferred intraperitoneally (week 0). Different letters (a, b, etc.) indicate statistical significance among means by one-way ANOVA and Tukey’s post-hoc comparison. Data are displayed as the mean ± SEM with *n* = 3 to 4 mice/time point.

In contrast to *Cd74*, *Cxcr4* mRNA expression (Figure 3) slightly increased at 1 week from induction (1.43-fold increase, *p* = 0.093) with minimal increase (*p* < 0.05) from weeks 2- and 4-weeks post-endometriosis induction (week 0) in mice with WT lesions. However, at week 8, *Cxcr4* expression was significantly less compared to all other time points (*p* < 0.05; Figure 3). 

Compared to WT lesions, lesions developed from *miR-451a* deficient tissue exhibited a significant increase at 1-week post-induction and this increase remained significantly elevated through week 8 post-induction. In comparison to *Cd74* levels, *Cxcr4* levels were approximately 30-fold less abundant in lesion tissue from WT mice and 180-fold less abundant in lesions derived from *miR-451a* deficient tissue.

## 4. Discussion

Experimental mouse models are essential for enhancing our understanding on endometriosis pathophysiology [5]. The mouse model which we initially described in 2011 [6] consists of using PMSG-primed endometrial tissue from immature, immune competent, reproductively intact donors. Using this model, physiological levels of endogenous E2 are liberated which aids in the establishment of ectopic lesions (endometrial tissue from either unstimulated or PMSG + hCG, the latter in which progesterone levels are elevated, do not establish ectopically). Using mature (2–4 months of age), immune competent, reproductively intact, non-hormonally stimulated host mice has several advantages. First, exogenous E2 is not provided allowing for normal hormonal changes which occur during a reproductive cycle, which more closely mimics that in patients. This in turn allows for examination of endpoints of interest under hormonal levels which are more representative compared to supraphysiological levels of E2. Second, host mice are immune competent which again more closely mimics the human peritoneal environment and allows for assessment of the potential role of immune/inflammatory mediators in endometriosis pathophysiology. Third, a known number of endometrial fragments of a known size are transferred to recipient mice allowing for assessment of lesion growth over time. Lastly, using this mouse model, we can assess lesion dynamics as lesions first establish (week 1) through growth (weeks 2 to 4) and eventual regression.

With this in mind, we used wild-type or *miR-451a* deficient tissue to establish lesions in wild-type hosts to assess the natural progression of lesion expression of the Mif receptors, Cd74 and Cxcr4 over an eight-week timespan. Using this model, we report for the first-time concurrent assessment of both *Cd74* and *Cxcr4* expression in endometriotic lesion tissue from mice with experimentally-induced endometriosis. CD74 was the first reported cell surface receptor for MIF [29]. Veillat and colleagues [25] first reported that MIF could induce expression of angiogenic factors in endometriotic stromal cells and this effect could be attenuated by knockdown of CD74 expression. Mahdian and associates reported elevated *MIF* and *CD74* mRNA expression in ectopic lesion tissue compared to eutopic endometrium from both control and patients with endometriosis [39], while we reported elevated CD74 protein expression and localization to glandular epithelium [30]. Thus, our observation of elevated *Cd74* expression in our mouse model in which ectopic lesions were derived from wild-type tissue supports these prior reports which utilized human tissues, validating this experimental model.

As mentioned earlier, CXCR4 is capable of forming dimers with MIF-bound CD74 [31,32,33,34]; however, the majority of the studies which have evaluated CXCR4 in the context of endometriosis have been evaluating it as a receptor for CXCL12 [35,36]. A recent report by Tal and colleagues [39] used Cxcr4-deficient tissue to establish experimentally-induced endometriosis and found that, compared to control lesions (which expressed Cxcr4), less lesions developed but development was not completely inhibited. This observation coupled with our current observation that *Cxcr4* expression is minimally elevated in experimental endometriosis may suggest that other receptors such as Cd74 may play a more prominent role in endometriotic lesions development and/or survival. Alternatively, other receptors capable of binding with MIF-CD74 complexes, such as CD44 [40,41,42] could also contribute to MIF signaling in endometriosis [43]. 

With respect to experimental endometriosis induced using *miR-451a* deficient tissue, we were intrigued to find augmented expression of *Cd74* compared to levels of expression in wild-type lesions. However, we are uncertain as to what the mechanism for this up-regulation may be. It is tempting to speculate that the elevated expression of *Cd74* may occur in response to the reduced levels of Mif ligand [20] in an attempt to maintain lesion survival. Accumulating data from our lab [18,20,44,45] support the notion that increased levels of endometriotic lesion *miR-451a* function to curtail lesion survival by at least impart reducing Mif expression but other indirect targets of *miR-451a* which impact lesion survival [44] may come into play as well. Alternatively, loss of *miR-451a* may lead to changes in other miRNAs which could lead to enhanced *Cd74* transcript expression. We recently reported that *miR-451a* decreases expression of a miRNA (*miR-25-3p*) in vitro which is a bonafide regulator of *PTEN* expression [45]. Thus, it may be likely that expression levels of *miR-451a* (either absence at the time of lesion establishment or elevation which occurs as lesions age) may influence the expression of other miRNAs which could impact *Cd74* expression. Clearly, more detailed assessment of the role of *miR-451a*, the MIF-pathway and other pathways modulated by *miR-451a* in the pathophysiology of endometriosis are warranted for study. 

In summary, using a reproductively intact, immune competent mouse model of experimental endometriosis, we report lesion expression of Mif receptors *Cd74* and *Cxcr4*. *Cd74* is robustly expressed, and its expression increases with lesion growth and survival, expressing a similar pattern to that observed in humans as well as in a non-human primate model of experimental endometriosis. Use of this mouse model can be used in future studies to further explore the pathophysiology of endometriosis in making bench to bedside contributions which will enhance our understanding on the pathophysiology of endometriosis.

## Figures and Tables

**Table 1:** Primer sequences used for qRT-PCR.

Gene (Accession Number)	Forward Primer	Reverse Primer
*mCD74* (NM_010545)	3′-GAAGCTTCCGAAATCTGCCA-5′	3′-CATTGGACGCATCAGCAAGG-5′
mCxcr4 (NM_009911)	3′-GGAACCGATCAGTGTGAGTATATA-5′	3′-CAGGGTTCCTTGTTGGAGTCA-5′
18S (X03205.1)	Thermo Fisher 4310893E	Thermo Fisher 4310893E

## Data Availability

Not applicable.
